# Chain Size and Knots of Ring Polymers in All-Crossing and Intra-Crossing Melts

**DOI:** 10.3390/polym17070854

**Published:** 2025-03-23

**Authors:** Jiangyang Mo, Jingqiao Guo, Xue Yu, Jianlei Yang, Guodong Hu, Jianhui Xin, Mengxia Yan, Yuan Wang, Yongjie Mo, Yuxi Jia, Lianyong Wu, Yongjin Ruan

**Affiliations:** 1 Shandong Key Laboratory of Biophysics, Institute of Biophysics, Dezhou University, Dezhou 253023, China; jymo@dzu.edu.cn (J.M.); y12180348@mail.ecust.edu.cn (X.Y.); yangjianleide@126.com (J.Y.); guodong.hu537@gmail.com (G.H.); jhxin_mxyan@163.com (J.X.); yanmengxia1219@163.com (M.Y.); jymosdu@163.com (Y.M.); 2 State Key Laboratory of Polymer Science and Technology, Changchun Institute of Applied Chemistry, Chinese Academy of Sciences, Changchun 130022, China; 3 School of Materials Science and Engineering, Shandong University, Jinan 250061, China; jia_yuxi@sdu.edu.cn; 4 Shandong Provincial Engineering Research Center of Novel Pharmaceutical Excipients and Controlled Release Preparations, College of Pharmacy, Dezhou University, Dezhou 253023, China; jymosdu@mail.ustc.edu.cn; 5 Qilu Synva Pharmaceutical Co., Ltd., Dezhou 253023, China; lianyong.wu@qilu-pharma.com

**Keywords:** ring polymers, interchain constraint, intrachain constraint, knot, Monte Carlo

## Abstract

Using dynamic Monte Carlo simulations based on the bond-fluctuation model, we systematically investigated the size and knots of ring polymers in all-crossing systems and intra-crossing systems. Our results demonstrate that the interchain constraint can increase the knotting probability, but does not alter the scaling relationship between knotting probability and chain length for ring polymers in melts. Having established that, we derived the interchain constraint contribution to the free energy of ring polymers in intra-crossing systems based on the knotting probability and obtained the scaling relationship between the size *R* and chain length *N*, i.e., *R*~*N*^1/6^. And, by calculating the mean-squared radius of gyration of ring polymers in intra-crossing systems, we validated these scaling results. Finally, we analyze the size of knotted ring polymers with different types and compare corresponding scaling exponents for size versus chain lengths of ring polymers with different knotting complexities. These results provide fundamental insights into the static properties of ring polymers in melts.

## 1. Introduction

The topological constraints of ring polymers play a pivotal role in their static and dynamic properties, including conformation, diffusion, and rheology [1,2,3]. Since Jacob and Wollman [4] first proposed the existence of circular chromosomes, and Freifelder et al. [5] obtained the visual cyclic structure of viral DNA, many studies on ring polymers have been carried out based on experiments [6,7,8,9,10,11], theories [12,13,14,15,16,17,18,19], and computer simulations [19,20,21,22,23,24,25,26,27,28,29,30,31]. However, some open questions still remain regarding ring polymers in melts.

For example, different from linear polymers, both experiments [7,8,9,10,11] and simulations [1,15,19,20,21,22,23,24] found the scaling exponent *ν* of the radius of gyration *R*_g_~*N^ν^* decreases from 1/2 to 1/3 with increasing chain length *N* for ring polymers in melts. Regarding the use of theories [11], the solution to the conformational problem for ring melts is hampered, because the Hamiltonian representation of the topological constraints has not been found. Two different approaches, geometry and free energy approaches, are used to assess the scaling relationship between *R*_g_ and *N* in ring melts. The first approach starts from the assumed geometric structures of ring polymers. The early lattice animal model with double folded loops [32] arrives at *R*_g_~*N*^1/4^, but the crumpled globule proposed by Grosberg et al. [33,34] claims that *ν* = 1/3 for collapsed long chains, and the ideal lattice trees or animals model [32,35] describing a ring polymer in a gel predicted *ν* = 1/3. Furthermore, Obukhov et al. [16] put forward the decorated loop model and provided an analytic expression for *R*_g_.

The second approach considers the heuristic expressions for the free energy in terms of the Flory-type and obtains the power law dependence of *R*_g_ from *N* by deriving and minimizing the free energy of ring polymers in melts. Cates and Deutsch [12] defined the free energy *F* of a ring polymer in melts as follows:

(1) $$F\sim k_{B}T\frac{R^{3}}{N}+k_{B}T\frac{N}{R^{2}}$$

where *k*_B_ represents the Boltzmann constant, *T* is temperature, and *R* is the size of the polymer ring, respectively. The first term of Equation (1) denotes the interchain constraint contribution (*F*_inter_) resulting from the nonconcatenation constraint with its neighbors, and the second term is the intrachain constraint contribution (*F*_intra_), representing the free energy required to force a Gaussian chain polymer into a region of size *R*. Upon the usual minimization of *F*, Cates and Deutsch obtained a scaling law *R*~*N^ν^* for *ν* = 2/5. Subsequently, Suzuki et al. [19] recalculated the interchain constraint contribution *F*_inter_ and reached *ν* = 1/3 for long ring polymers in melts. Moreover, Sakaue [17,18] took into account many-body effects of topological origin in dense systems and obtained *ν* = 1/2 for short ring polymers and the broad crossover from 2/5 to 1/3 for relative long ring polymers. Furthermore, beginning with considering an isolated ring in a linear melt, Lang et al. [15,36] argued that the free energy of intrachain constraint contribution, *F*_intra_, is composed by the entropy loss upon squeezing an ideal ring and topological nonconcatenation contribution. On the basis of above discussion, Lang et al. [15,36] predicted the scaling exponents as 2/5, 3/8, and 4/9 for ring polymers in melts with short lengths, intermediate lengths, and long lengths, respectively. The results of the above heuristic expressions and scaling exponents are summarized in Table 1. However, the Flory-type expressions for interchain constraint contributions and intrachain constraint contributions have not been confirmed by experiments or simulations.

Knotting is a common phenomenon that occurs in biological and chemical systems and plays a significant role in slowing down the relaxation of a compressed DNA [37], facilitating catalysis of proteins [38], jamming nanopore translocation [39], and reducing the mechanical strength of polymers [40]. It has been shown that the knotting probability *P*(*N*) of ideal and self-avoiding ring polymers approaches unity with an exponential form [41,42,43] as follows:

(2) $$P(N)\sim 1-C\exp(N/N_{0})$$

where *C* is a constant, and *N*_0_ is the characteristic length of random knotting. In our previous study [31], we reported the size and dynamics of ring polymers under different topological constraints by tuning interchain and intrachain interactions independently, and introduced four ring polymer systems, i.e., non-crossing systems, all-crossing systems, inter-crossing systems, and intra-crossing system. The knotting probability of ring polymers in an intra-crossing system, where the chain uncrossability constraint is always applied for interchain movements, but is never applied for intrachain movements, has not been investigated.

In this work, following our previous work [31], we systematically investigate the knotting probability and their size of ring polymers in an all-crossing system and intra-crossing system and discuss the heuristic expressions for the free energy of ring polymers in melts using Shaffer’s dynamic Monte Carlo (MC) simulation [44,45] with the bond fluctuation model. In the following section, we present our simulation model and method. In the Results and Discussion, the simulation results are discussed based on three aspects, followed by the overall conclusions drawn from these results, which are presented in Section 4.

## 2. Simulation Method and Model

### 2.1. Simulation Method

Compared to Carmesin and Kremer’s original bond fluctuation model [46], Shaffer’s [44,45] is more convenient with regard to turning the topological interaction on and off by controlling bond crossing, and is faster in obtaining equilibrium configurations. The model has been extensively applied to polymer melts [19,21,29,47,48] and polymer blends [49,50,51,52,53] due to its computational efficiency. In this model, all polymers are placed on a 3D cubic lattice with a size *L*, and periodic boundary conditions are applied in all coordinate directions. To avoid the finite size effect, the length of the simulation box was set to *L* > 3*R*_g_ for the respective ring polymer chains. The occupancy of the lattice is fixed at 0.5, which has been shown to be sufficiently high to describe melt-like systems [54]. Using the same occupancy fraction, our previous work [31] also agrees with the results of the molecular dynamics simulation [1,2], such as the scaling exponent of *N* for *R* decreasing from 2/5 to 1/3 with increasing *N,* and the scaling relation being *g*_3_(*t*)~*t*^0.75^ for ring polymers in real systems, where *g*_3_(*t*) represents the mean-square displacement at time *t*.

The initial configurations of ring polymers are placed on the lattice by double folding and each ring is isolated from the others to avoid self-knots and concatenations. To equilibrate these highly ordered initial configurations, we run *τ*_eq_ Monte Carlo steps (MCSs), and then we run another *τ*_run_ MCSs to collect and analyze data; all data reported are averages obtained over four independent simulations. The detailed simulation parameters are listed in Table S1 of the Supplementary Materials. Herein, a MCS is defined as a trial event for attempting to move every monomer to a randomly chosen nearest-neighbor site in the box once, and the displacement is accepted only if it satisfies the excluded volume constraint, chain connectivity constraint, and bond uncrossability constraint. The excluded volume constraint is implemented by allowing only a single monomer to occupy one lattice site simultaneously, and the chain connectivity constraint is enforced by restricting the length of the bond connecting two neighboring monomers to the set of values $\left\{1,\;\sqrt{2},\;\sqrt{3}\right\}$ in units of lattice spacing. The bond uncrossability constraint is switched off for ring polymers in all-crossing systems, while the uncrossability constraint is always applied for interchain movements, but is never applied for intrachain movements for ring polymers in intra-crossing systems. To ensure that the configurations of the ring are in equilibrium, the *τ*_eq_ and *τ*_run_ of other simulation systems are 15 times larger than the relaxation time of the corresponding ring polymers.

The ring polymer size is characterized by the mean-square radius of gyration $\langle R_{g}^{2}\rangle$, which is defined by

(3) $$\langle R_{g}^{2}\rangle =\frac{1}{N^{2}}\sum_{i=1}^{N}\sum_{j=i}^{N}{\left(R_{i}-R_{j}\right)}^{2}$$

where *R_i_* is the position of monomer *i*, and ⟨⋯⟩ indicates the ensemble average over all available conformations of ring polymers.

### 2.2. Identification of Knots

**Figure 1:**
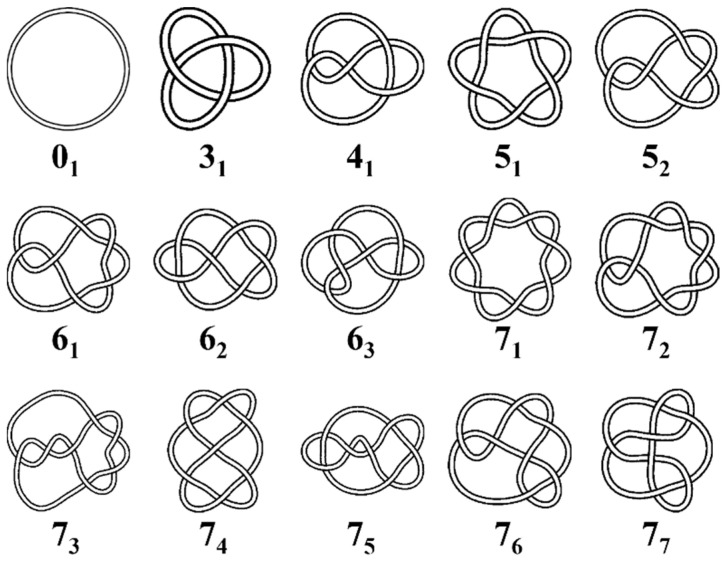
Schematic illustration of unknot (the trivial knot, 0_1_) and the prime knots with up to seven minimal crossings. In the notation, the first number indicates the minimal number of crossings a given knot can have in a standard projection and the second number indicates its tabular position among the knots with the same number of crossings in standard tables of knots [55].

As shown in Figure 1, knots are usually categorized according to the minimum number of crossings in a projection onto a plane, and different knot types have different Alexander polynomials [55], except that occasionally multiple knot types share the same Alexander polynomial. Thus, we identify the knot types by the calculation of Alexander polynomials. We tabulate the Alexander polynomials for 250 knot types with 0 ≤ *N*_cross_ ≤ 10, where *N*_cross_ represents the minimal crossing number of the knot. Then, for a given ring polymer conformation, we project the conformation on a plane, calculate the Alexander polynomial, and obtain the knot type through the mapping between Alexander polynomials and knot types. It is worth noting that some knots share the same Alexander polynomial, such as composite knots 3_1_#3_1_ and 3_1_#4_1,_ which share the same Alexander polynomials with 8_20_ and 8_21_ knots, respectively. [56] Because these overlapping Alexander polynomials usually correspond to the composite knots [57] and the probability of the overlapped Alexander polynomial between the 3_1_#3_1_ and 8_20_ knots is close to the square of the probability of the 3_1_ knot [58], it can be said that the overlapped Alexander polynomial comes from the 3_1_#3_1_ knot. We assign these overlapped Alexander polynomials to composite knots. For very complex knots with more than 10 crossings, it is impractical to determine the knot type using the Alexander polynomial, so we do not classify the knots with more than 10 crossings, and treat these knots as one category.

## 3. Results and Discussion

### 3.1. Knotting Probability of Ring Polymers

**Figure 2:**
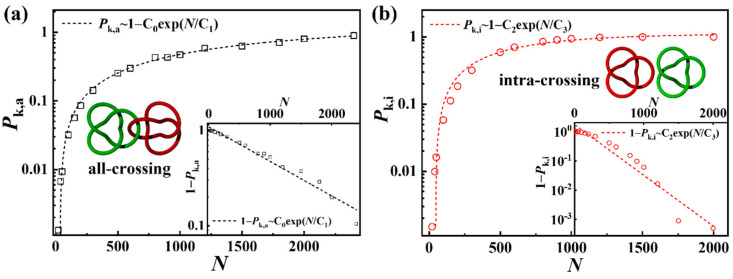
Knotting probability of ring polymers in (**a**) all-crossing systems, *P*_k,a_, and (**b**) intra-crossing systems, with *P*_k,i_ versus chain length; the insets show the relationship between the unknotting probability and chain length. And, the schematics illustrate the model of ring polymers in (**a**) all-crossing systems and (**b**) intra-crossing systems.

Knotting probability and properties of all-crossing ring polymers have been studied extensively in recent decades [41,42,43,59]. We compared the knotting probability of ring polymers in all-crossing systems and intra-crossing systems. As shown in Figure 2a, the knotting probability of a ring polymer in all-crossing systems follows *P*_k,a_~1 − C_0_exp(*N*/C_1_), where C_0_ and C_1_ are constants and C_0_ = 1.1418, and C_1_ = −2217.58. The inset in Figure 2a illustrates the linear relationship between 1 − *P*_k,a_ and C_0_exp(*N*/C_1_) more clearly, and this result is consistent with Equation (2). However, as shown in Figure 2b, the knotting probability of ring polymers in intra-crossing systems is *P*_k,i_~1 − C_2_exp(*N*/C_3_), where C_2_ and C_3_ are constants and C_2_ = 1.1168 and C_3_ = −597.74, which also corresponds to Equation (2). The inset of Figure 2b confirms the linear relationship between 1 − *P*_k,i_ and C_2_exp(*N*/C_3_). The result indicates that the interchain constraint has no influence on the scaling relationship between knotting probability and chain length of the ring polymers, but observably increases the knotting probability.

### 3.2. Interchain Constraint Contribution to the Free Energy

**Figure 3:**
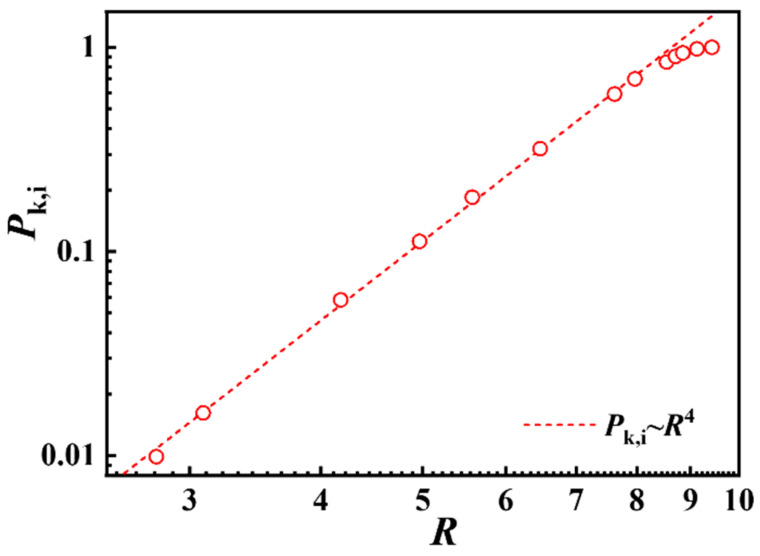
Scaling relation of knotting probability *P*_k,i_ with chain size *R* for ring polymers in intra-crossing systems.

As shown in Table 1, based on different assumptions, many researchers have put forward their heuristic expressions for interchain constraint contribution (*F*_inter_) and intrachain constraint contribution (*F*_intra_) of ring polymers in melts. Since there is no interchain or intrachain constraint in all-crossing systems, we focus on the intra-crossing systems to calculate the interchain constraint contribution which is required to squash a Gaussian chain of *N* steps into a region of linear size *R* [12]. Figure 3 gives the scaling relationship between knotting probability *P*_k,i_ and chain size *R* for ring polymers. The same results of all-crossing systems are given in Figure S1 of the Supplementary Materials. As shown in Figure 3, knotting probability *P*_k,i_~*R*^4^ for ring polymers in intra-crossing systems, which indicates a scaling of interchain constraint free energy *F*_inter_~*k*_B_*TR*^4^. The intrachain constraint of a ring polymer mainly comes from its neighboring ring polymers; according to the theoretical result of Cates et al. [12] and Suzuki et al. [19], the intrachain constraint free energy can be represented by *F*_intra_~*k*_B_*TN*/*R*^2^. Therefore, we obtained the free energy for ring polymers in intra-crossing systems as

(4) $$F\sim k_{B}TR^{4}+k_{B}T\frac{N}{R^{2}}$$

which, following the usual minimization, yields

(5) $$R\sim N^{1/6}$$

the scaling relation in Equation (5) has been verified by our previous study [31], which confirms our expression for the free energy of ring polymers in intra-crossing systems shown in Equation (4).

**Figure 4:**
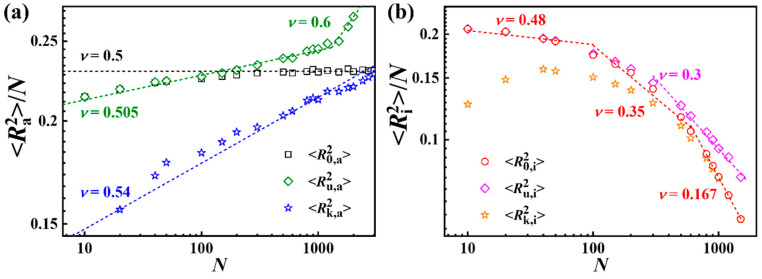
Normalized mean-squared radius of gyration versus chain length for knotting and unknotting ring polymers. $\langle R_{0,a}^{2}\rangle$, $\langle R_{u,a}^{2}\rangle$, and $\langle R_{k,a}^{2}\rangle$ represent the mean-squared radii of gyration for all rings, unknotting rings, and knotting rings in (**a**) all-crossing systems, respectively. $\langle R_{0,i}^{2}\rangle$, $\langle R_{u,i}^{2}\rangle$, and $\langle R_{k,i}^{2}\rangle$ represent rings in (**b**) intra-crossing systems. The error bars are smaller than the sizes of the symbols.

Moreover, we calculated the mean-square radius of gyration of knotting ring polymers and unknotting ring polymers in all-crossing systems and intra-crossing systems, respectively. As shown in Figure 4a, the power law relationship $\langle R_{0,a}^{2}\rangle \sim N^{2\nu }$, for which the scaling exponent ν = 0.5 was established, is in accordance with ideal chains [31]. The value of $\langle R_{0,a}^{2}\rangle$ equals the mean-squared radius of the gyration of unknotting ring polymers $\langle R_{u,a}^{2}\rangle$ for short chain lengths. With an increase in chain length *N*, the value of $\langle R_{0,a}^{2}\rangle$ becomes closer and closer to that of the knotting ring polymers $\langle R_{k,a}^{2}\rangle$. The reason is that the number of knotting rings increases with the increase in N, as shown in Figure 2a, which also results in the value of $\langle R_{0,a}^{2}\rangle$ being close to the value of $\langle R_{u,a}^{2}\rangle$ for short ring chains, and approximating to the value of $\langle R_{k,a}^{2}\rangle$ for long ring chains. Furthermore, the scaling exponent of $\langle R_{u,a}^{2}\rangle \sim N^{2\nu }$ increases with increasing *N*.

In contrast to all-crossing systems, the power law relationship between the mean-square radius of gyration and the chain length is smaller than 0.5 due to the interchain constraint. As shown in Figure 4b, the scaling exponent of *N* for $\langle R_{k,a}^{2}\rangle$ decreases from 0.48 to 0.167 with increases in *N*, which is consistent with our theoretical prediction in Equation (5). And, the value of $\langle R_{0,i}^{2}\rangle$ overlaps with the mean-squared radius of gyration of unknotting ring polymers $\langle R_{u,i}^{2}\rangle$ for short chain lengths, and approaches that of the knotting ring polymers $\langle R_{k,i}^{2}\rangle$ for large *N* values.

### 3.3. Probability and Sizes of Different Types of Knotting Rings

**Figure 5:**
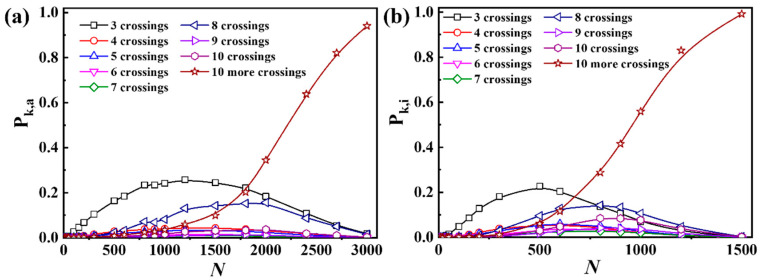
Knotting probability versus chain length for ring polymers with different complexities in (**a**) all-crossing systems and (**b**) intra-crossing systems. Different symbols represent different crossing numbers of knotting types in ring polymers.

To identify the complexity of knotting ring polymers in melts, we calculated the knot type of ring polymers with different chain lengths and topological constraints. As shown in Figure 5, short ring polymers tend to form the knotting configuration with 3 crossings. And with increasing chain length, the probability of knots with less than 10 crossings first increases and then decreases in both all-crossing systems and intra-crossing systems, while the probability of knots with more than 10 crossings monotonically increases. These results demonstrate the rapid increase in complexity of knots with chain length in all-crossing systems and intra-crossing systems. Moreover, the peak of the knotting probability curve for ring polymers in all-crossing systems appears much later than those in intra-crossing systems, indicating the interchain topological constraints could increase the probability of knotting.

**Figure 6:**
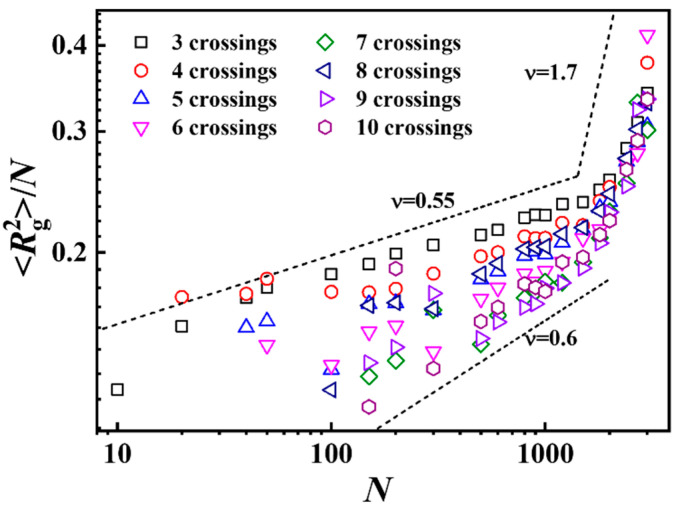
Normalized mean-squared radius of gyration $\langle R_{g}^{2}\rangle /N$ versus *N*, with different complexity in all-crossing systems. Different symbols represent different numbers of crossings in ring polymer knotting. The error bars are smaller than the sizes of the symbols.

Figure 6 shows the scaling relationship between the mean-squared radius of gyration versus *N* for ring polymers with different complexities in all-crossing systems. Different from the scaling exponent, *ν* = 0.54 of *N* for the average size of all knotting ring polymers shown in Figure 4a; the scaling exponents for the mean-squared radius of gyration versus the chain length vary from 0.55 to 0.6 for ring polymers with different knotting complexities in all-crossing systems when the chain length is smaller than 1000. Based on these results, we can speculate that the scaling exponents for knotting types with 10 or more crossings are much smaller than 0.54. With further increases in chain length, the scaling exponents rapidly increase to 1.7 with different knotting complexities. Moreover, for a given chain length, the higher the number of crossings of the knotting polymers, the smaller the size of the polymers.

**Figure 7:**
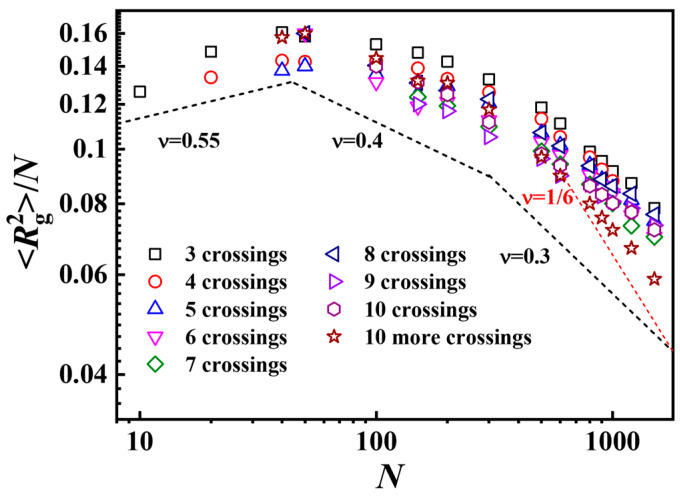
Normalized mean-squared radius of gyration versus *N* for ring polymers with different complexities in intra-crossing systems. Different symbols represent different numbers of crossings in knotting ring polymers. The error bars are smaller than the sizes of the symbols.

The scaling relationship between the mean-squared radius of gyration versus *N* for ring polymers with different complexities in intra-crossing systems is shown in Figure 7. The scaling exponents decrease from 0.55 to 0.3 with the increase in chain length for ring polymers with different knotting complexities in intra-crossing systems, with a similar trend in the average size of all knotting ring polymers, shown in Figure 4b. Quantitatively, the value of the scaling exponents for ring polymers with different knotting complexities is larger than that for the average size of all knotting ring polymers. Moreover, the higher the number of crossings in the knotting polymers, the smaller the size of the polymers with same chain length, which is in agreement with the results for ring polymers in all-crossing systems.

**Figure 8:**
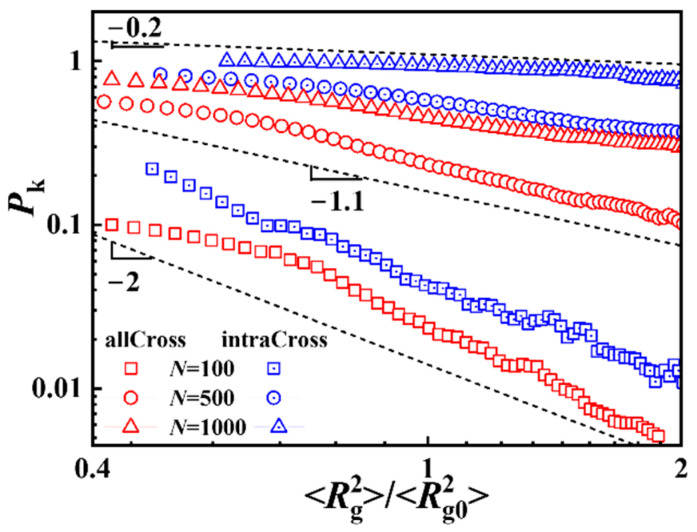
Knotting probability *P*_k_ versus the ratio of the mean-squared radius of gyration for knotting rings to those of the average value for all ring polymers.

Finally, we calculated the knotting probability of ring polymers with different sizes. As shown in Figure 8, the knotting probability decreases with increases in size for ring polymers with same chain length, and the knotting probability of ring polymers in intra-crossing systems is significantly larger those in all-crossing systems, which agrees with the results shown in Figure 5. Furthermore, ring polymers, both in all-crossing systems and intra-crossing systems, present a good scaling relationship between knotting probability and the ratio of size for knotting rings to size for the average value of all ring polymers. For short ring polymers (*N* = 100), the scaling exponent is close to two, and the ring polymer is hardly knotted when its size is smaller than the average size of all ring polymers. For relatively long ring polymers (*N* = 500 and 1000), the scaling exponent decreases obviously and the knotting probability is nearly independent of the size for ring polymers with *N* = 1000 in intra-crossing systems, since almost all of the polymers are knotted.

## 4. Conclusions

By employing dynamic MC simulations of the bond-fluctuation model along with an analysis of knotting type, we systematically investigated the size and knots of ring polymers in all-crossing systems and intra-crossing systems.

First, we calculated the knotting probability of ring polymers and found that the interchain constraint can lead to higher knotting probability, but has no influence on the scaling relationship between knotting probability and the chain length of ring polymers. Then, we derived the interchain constraint contribution to the free energy of ring polymers in intra-crossing systems based on the knotting probability. According to the free energy, we obtained the scaling relationship between the size and chain length, i.e., *R*~*N*^1/6^, and we further verified this result by calculating the mean-squared radius of gyration of the ring polymers in intra-crossing systems. Finally, we analyzed the size of knotted ring polymers of different types, and found that the scaling exponents for size, versus the chain length of ring polymers with different knotting complexities, is larger than the scaling exponents of average knotting ring polymers in all-crossing systems, whereas the scaling exponents of ring polymers with different knotting complexities have similar trends with the average size of all knotting ring polymers.

The analyses presented in this work provide a better understanding of the static properties of ring polymers in melts, and the results contribute to calculations of the free energy of real ring polymer systems.

## Figures and Tables

**Table 1:** Heuristic expressions for the free energy of ring polymers in melts and the scaling exponents between the chain size *R* and chain length *N*.

	Interchain Constraint Contribution *F*_inter_	Intrachain Constraint Contribution *F*_intra_	Scaling Exponents *ν*
Cates and Deutsch [12]	$k_{B}T\frac{R^{3}}{N}$	$k_{B}T\frac{N}{R^{2}}$	2/5
Suzuki et al. [19]	$\frac{k_{B}T}{NR^{2}}{\left(\frac{4\pi }{3}R^{3}-N\right)}^{2}$	$k_{B}T\frac{N}{R^{2}}$	1/3
Sakaue [17,18]	$-k_{B}Tln(1-\frac{R^{3}}{N})$	$k_{B}T\frac{N^{3}}{R^{6}}$	1/2 2/5~1/3
Lang et al. [15,36]	$k_{B}TR^{2}$	$k_{B}T\frac{N}{R^{2}}+k_{B}T\frac{N^{2}}{R^{3}}$	2/5
	$k_{B}TR^{2}$	$k_{B}T\frac{N}{R^{2}}+k_{B}T\frac{N^{3}}{R^{6}}$	3/8
	$k_{B}T\frac{R^{3}}{N}$	$k_{B}T\frac{N}{R^{2}}+k_{B}T\frac{N^{3}}{R^{6}}$	4/9

## Data Availability

Data are contained within the article.

## Supplementary Materials

The following supporting information can be downloaded at: https://www.mdpi.com/article/10.3390/polym17070854/s1, The detailed simulation parameters are listed in Table S1, and the scaling relation of knotting probability *P*_k,a_ with chain size *R* for ring polymers in all-crossing systems is shown in Figure S1 in the Supplementary Materials.
