# Supplementary material for: Chain Size and Knots of Ring Polymers in All-Crossing and Intra-Crossing Melts
Source: Polymers (Basel). 2025 Mar 23;17(7):854. doi: 10.3390/polym17070854 (PMC11991610; doi:10.3390/polym17070854)
Supplement: Supplementary file 1 [file polymers-17-00854-s001.zip › polymers-3530513-supplementary.pdf]

Supplementary Materials:

"Chain Size and Knots of Ring Polymers in All-crossing and Intra-crossing Melts"

Jiangyang Mo [1,2,3], Jingqiao Guo [4,*], Xue Yu [1], Jianlei Yang [1], Guodong Hu [1], Jianhui Xin [1], Mengxia Yan [1], Yuan Wang [4], Yongjie Mo [1], Yuxi Jia [3], Lianyong Wu [5], Yongjin Ruan [2,*]

[1] Shandong Key Laboratory of Biophysics, Institute of Biophysics, Dezhou University, Dezhou 253023, China
[2] State Key Laboratory of Polymer Science and Technology, Changchun Institute of Applied Chemistry, Chinese Academy of Sciences, Changchun 130022, China
[3] School of Materials Science and Engineering, Shandong University, Jinan 250061, China.
[4] Shandong Provincial Engineering Research Center of Novel Pharmaceutical Excipients and Controlled Release Preparations, College of Pharmacy, Dezhou University, Dezhou 253023, China
[5] Qilu Synva Pharmaceutical Co., Ltd. , Dezhou 253023, China
* Correspondence: guojingqiao@dzu.edu.cn (J.G.); yjruan@ciac.ac.cn (Y.R.)

Table S1. Simulation parameters for different chain lengths N of the two systems. L_{box}, τ_{eq} and τ_{run} are the cubic simulation box size, the equilibration time, and the time used for collecting data, respectively.

N	L_{box}	all-crossing		intra-crossing	
		τ_{eq}	τ_{run}	τ_{eq}	τ_{run}
10	20	5.0×10^4	5.0×10^4	5.0×10^4	5.0×10^4
20	20	8.0×10^4	8.0×10^4	8.0×10^4	8.0×10^4
40	20	1.0×10^5	1.0×10^5	1.0×10^5	1.0×10^5
50	20	2.0×10^6	2.0×10^6	2.0×10^6	2.0×10^6
100	20	2.0×10^6	2.0×10^6	2.0×10^6	2.0×10^6
150	30	2.0×10^6	4.0×10^6	2.0×10^6	4.0×10^6
200	40	1.0×10^7	8.0×10^6	1.0×10^7	8.0×10^6
300	30	1.5×10^7	1.5×10^7	2.0×10^7	2.0×10^7
500	40	2.5×10^7	$1..0\times10^8$	3.0×10^7	1.0×10^8
600	60	6.0×10^7	5.0×10^7	6.0×10^7	5.0×10^7
800	60	1.0×10^8	6.0×10^7	1.0×10^8	6.0×10^7
900	60	1.0×10^8	9.0×10^7	1.0×10^8	9.0×10^7
1000	60	1.0×10^8	1.0×10^8	2.0×10^8	1.0×10^8
1200	60	8.0×10^7	1.0×10^8	8.0×10^7	1.0×10^8
1500	60	8.0×10^7	1.5×10^8	8.0×10^7	2.0×10^8
1800	60	8.0×10^7	2.0×10^8	8.0×10^7	2.0×10^8
2000	60	1.0×10^8	2.5×10^8	1.0×10^8	3.0×10^8
2400	60	1.5×10^8	4.0×10^8	2.0×10^8	4.0×10^8

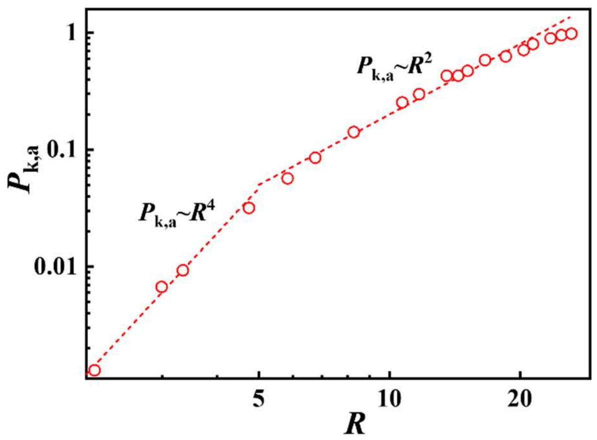

Figure S1. Scaling relation of knotting probability $P_{k,a}$ with chain size R for ring polymers in all-crossing systems.

As shown in Figure S1, the scaling exponent of R for $P_{k,a}$ decreases from 4 to 2 with the increasing R for ring polymers in all-crossing systems.
